# Supplementary material for: GmANKTM21 Positively Regulates Drought Tolerance and Enhanced Stomatal Response through the MAPK Signaling Pathway in Soybean
Source: Int J Mol Sci. 2024 Jun 26;25(13):6972. doi: 10.3390/ijms25136972 (PMC11241039; doi:10.3390/ijms25136972)
Supplement: Supplementary file 1 [file ijms-25-06972-s001.zip › ijms-3029266-supplementary.pdf]

Table S1: Primers Sequence

Primer name	Sequence (5'-3')	Applications
GmANKTM21-F	CGGGATCCATGACGATAGGATGAAG	Gene cloning
GmANKTM21-R	TTCGAGCTCTTAGCAGATGACACAGC	
GmANKTM21-RT-F	ATGAAGGGCATGGCAAAATC	Quantitative fluorescence PCR analysis
GmANKTM21-RT-R	TGGCCGAAATACAGACCTAC	
GmSPK2-RT-F	TTGAGGCTGAAAGAAGGGGT	
GmSPK2-RT-R	GAGAGGGAATTTAGCCAACCAC	
GmSPK4 RT-F	CTGCACTCACGACCCAAATCA	
GmSPK4 RT-R	CTAGGGTCATCCTGGTCCTCA	
GmSnRK2.6-RT-F	TGCAAAATGATGATGTGAATGACG	
GmSnRK2.6-RT-R	GCACAGACAAAATCACCGCTT	
GmABI5 RT-F	TGGCTATGCGGCAGTGAGTAAT	
GmABI5 RT-R	CTCTCCACCACCTTCTCCACAG	
GmABF4 RT-F	TTGTAAGTTTGTCTTGTTGAGGTTG	
GmABF4 RT-R	CTTGCCTAACAAAAGGGGTAGTGGT	
GmCYP707A1 RT-F	GCCGTTTGATCACCACTTTCC	
GmCYP707A1 RT-R	CAATCATCTTCCTCTGCCTCCTG	
GmTUA5-RT-F	TGCCAGTTCCCGTGCTTGAA	Amplification of internal reference genes
GmTUA5-RT-R	CTGCACCATCGTCAACCACTA	
